# Supplementary material for: P2X7 Receptors and TMEM16 Channels Are Functionally Coupled with Implications for Macropore Formation and Current Facilitation
Source: Int J Mol Sci. 2021 Jun 18;22(12):6542. doi: 10.3390/ijms22126542 (PMC8234106; doi:10.3390/ijms22126542)
Supplement: Supplementary file 1 [file ijms-22-06542-s001.zip › ijms-1245268-supplementary.pdf]

Supporting information

P2X7 receptors and TMEM16 channels are functionally coupled with implications for macropore formation and current facilitation

Kate Dunning[1¶], Adeline Martz[1¶], Francisco Andrés Peralta[1,2], Federico Cevoli[1], Eric Boué-Grabot[3,4], Vincent Compan[5,6], Fanny Gautherat[1], Patrick Wolf[1,7], Thierry Chataigneau[1], Thomas Grutter[1,2*]

[1]Université de Strasbourg, Centre National de la Recherche Scientifique, CAMB UMR 7199, Strasbourg, France.

[2]University of Strasbourg Institute for Advanced Studies (USIAS), Strasbourg, France.

[3]Université de Bordeaux, Institut des Maladies Neurodégénératives, UMR 5293, Bordeaux, France.

[4]CNRS, Institut des Maladies Neurodégénératives, UMR 5293, Bordeaux, France.

[5]Institut de Génomique Fonctionnelle (IGF), Université de Montpellier, CNRS, INSERM, Montpellier, France.

[6]Laboratoire d'Excellence Canaux Ioniques d'Intérêt Thérapeutique (LabEx ICST), Montpellier, France.

[7]Centre National de la Recherche Scientifique, Université de Strasbourg, Institut des Neurosciences Cellulaires et Intégratives, UPR-3212, Strasbourg, France.

[*]Corresponding author. E-mail: grutter@unistra.fr (TG)

¶These authors contributed equally to this work.

This PDF file includes:

Figures S1 to S5

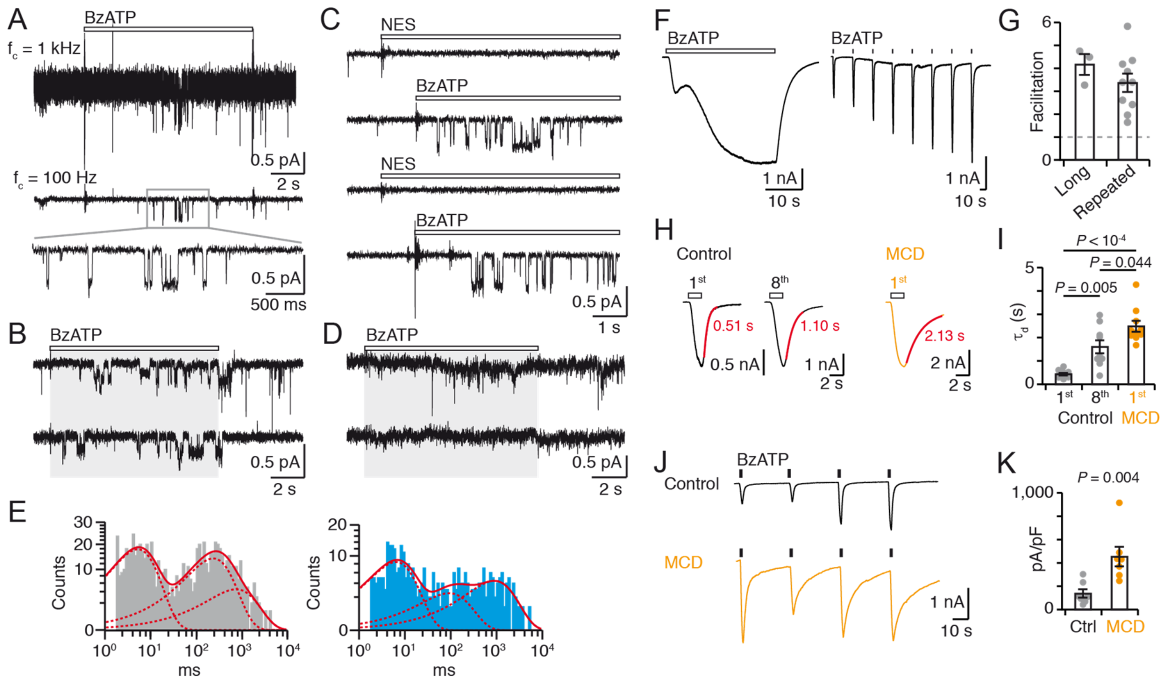

Fig. S1. Whole-cell and single-channel studies of P2X7: controls and additional data. (A) Full recording of a sweep showing single-channel currents evoked by 10 µM BzATP (duration of application indicated by the white bar) from an outside-out patch of HEK293T cell transiently transfected with rP2X7. Data were acquired at 10 kHz and filtered at a corner frequency (f_c) of either 1 kHz (upper trace) or 100 Hz (lower trace). Inset shows an expanded view of the recording filtered at 100 Hz. (B) Another example of single-channel recording (10 µM BzATP). Note that currents disappear upon washout. Shown sweeps were sequentially recorded from the same patch. Duration of application is indicated by the white bar and grey shading. (C) BzATP (10 µM), but not reduced divalent cation NES, elicits single-channel activity in an outside-out patch of rP2X7-expressing HEK293T cell. Shown sweeps were sequentially recorded from the same patch, in which NES and BzATP were alternatively applied (NES: Normal extracellular solution). (D) No single-channel

events (representative from 4 tested cells) are observed in response to BzATP perfusion in an outside-out patch excised from non-transfected cell. Shown sweeps were sequentially recorded from the same patch. (E) Closed dwell-time histograms for control (grey) and facilitated (blue) conditions, fitted by the sum (solid red line) of several exponential functions (dashed red lines). For control patches, τ_1 = 5.2 ms, a_1 = 0.49; τ_2 = 237 ms, a_2 = 0.39; τ_3 = 746 ms, a_3 = 0.12; mean closed time τ_C = 185 ms. For facilitated patches, τ_1 = 6.4 ms, a_1 = 0.55; τ_2 = 92 ms, a_2 = 0.16; τ_3 = 899 ms, a_3 = 0.29; τ_C = 279 ms. (F) Example of traces recorded from a rP2X7-transiently expressing HEK293T cell, showing the effect of facilitation on whole-cell currents from a sustained, long application of 30 s (*left*) or from repeated 2 s applications of 10 μM BzATP (*right*). (G) Summary of current facilitation determined between the end and beginning of long application or between the 8th and 1st application. (H) Expanded views of currents, shown in panel G, induced by the first and eighth application of BzATP in untreated (black traces) or MCD-treated HEK293T cells (orange trace). Deactivation currents were fit by single exponentials (red traces) and time constant of deactivation (τ_d) values are indicated. (I) Summary of time constant determined from first and eighth application in untreated cells and first application in MCD-treated cells (n = 9 cells for untreated condition and 10 cells for MCD-treated condition). *P* values are from paired Student's *t*-test (between first and eighth application data in control) and from Mann-Whitney test (between MCD and control data). (J) Typical whole-cell currents evoked by repeated 2 s applications of 10 μM BzATP in rP2X7-transiently expressing HEK293T cells either untreated (black trace) or treated by MCD (orange trace). (K) Summary of current density determined from first application in control cells and in MCD-treated cells (n = 7 cells for both conditions). *P* value is from unpaired

Student's *t*-test. Single-channel and whole-cell data were recorded at -120 and -60 mV, respectively. Single-channel data were filtered at a final f_c of 100 Hz unless specified.

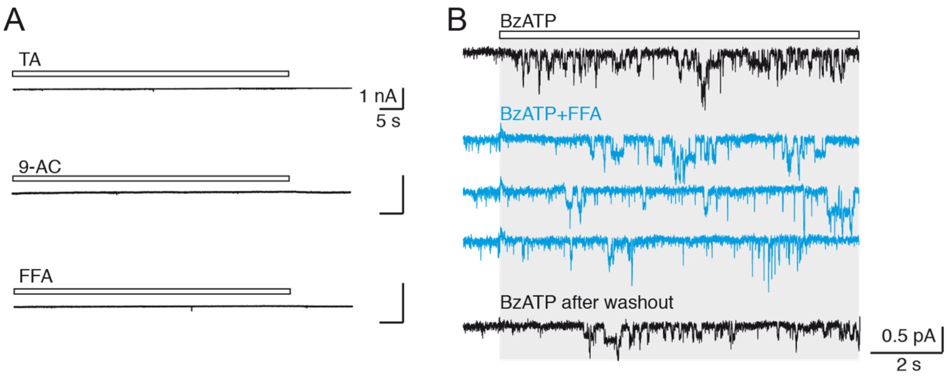

Fig. S2. Whole-cell and single-channel recordings during CaCC inhibitor application. (A) Application of 20 µM TA (duration of application indicated by the white bar), 1 mM 9-AC or 100 µM FFA for 60 s does not induce whole-cell currents in HEK293T cells transiently transfected with rP2X7. Data were recorded at -60 mV. (B) Single-channel rP2X7 currents elicited by 10 µM BzATP from an outside-out patch of HEK293T cells (black traces) are partially inhibited by 100 µM FFA when co-applied with 10 µM BzATP (BzATP+FFA, blue traces). Shown sweeps were sequentially recorded from the same patch. Note that inhibition is reversible after washout. Duration of application is indicated by the white bar and grey shading. Data were recorded at -120 mV, and filtered at a final f_c of 100 Hz.

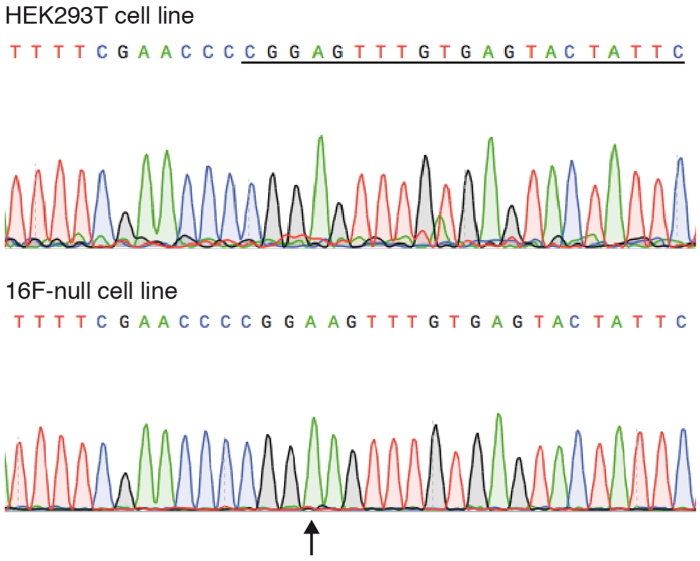

Fig. S3. CRISPR/Cas9 knockout of *TMEM16F* by a one nucleotide insertion. Sequencing chromatogram of the exon 2 region of endogenous *TMEM16F* genomic DNA in HEK293T cell line (upper) and 16F-null cell line (lower) where one nucleotide (A, indicated by black arrow) has been inserted, creating an erroneous frameshift mutation. The underlined sequence in HEK293T cell line indicates the sgRNA target sequence.

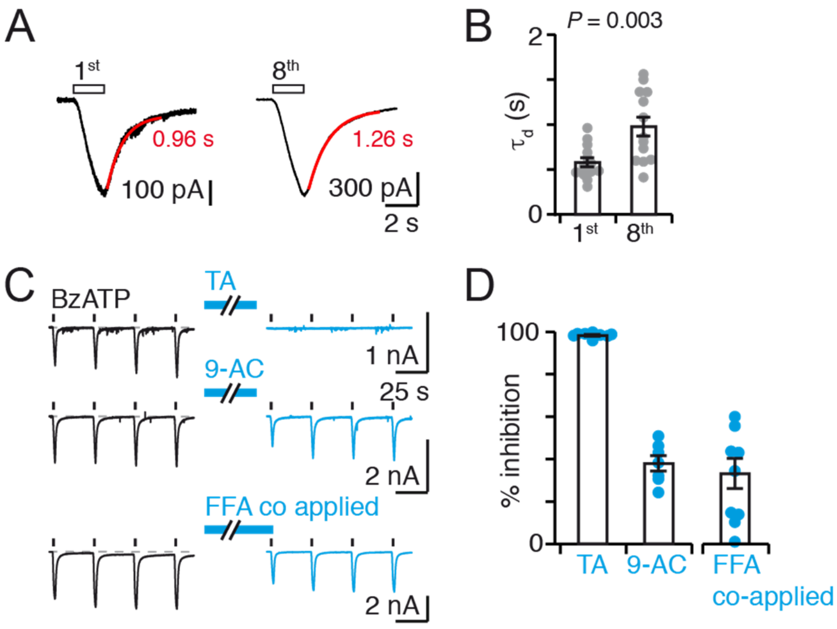

Fig. S4. Deactivation kinetics and CaCC inhibition in 16F-null cells. (A) Expanded views of currents induced by the first and eighth application of BzATP in 16F-null cells transiently expressing rP2X7. Deactivation currents were fit by single exponentials (red traces) and time constant of deactivation (τ_d) values are indicated. (B) Summary of time constant (n = 13 cells). *P* value is from paired Student's *t*-test. (C) Whole-cell currents evoked by repeated 2 s applications of 10 μM BzATP before and after perfusion of TMEM16 inhibitors in 16F-null cells transiently expressing rP2X7 using the same protocol as described in Fig 2A. (D) Summary of inhibition calculated as in Fig. 2B (n = 8 cells for TA, 7 for 9-AC, and 9 for FFA co-applied). Bars represent mean ± SEM. Data were recorded at -60 mV.

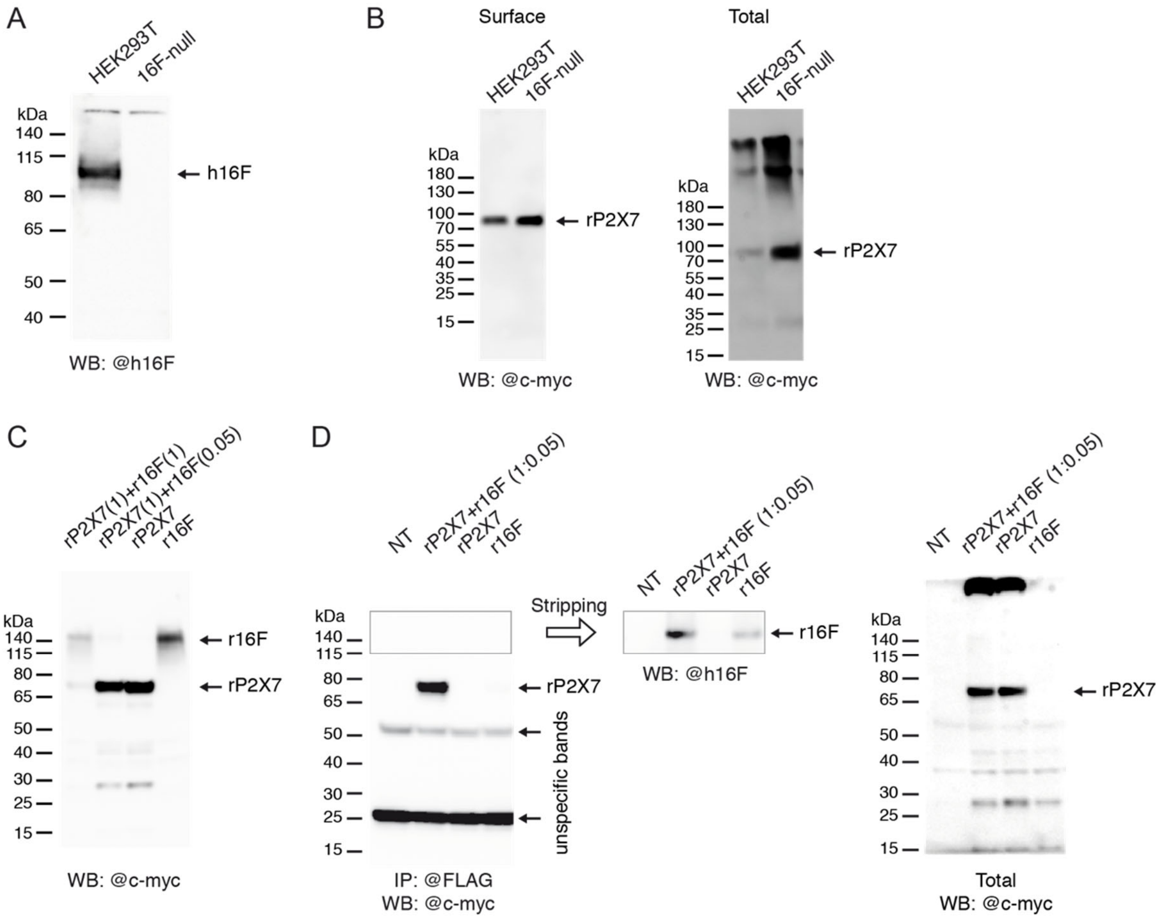

Fig. S5. Uncropped blots related to Figures 5 and 6. (A) Uncropped blot related to Fig. 5A. (B) Uncropped blots related to related to Fig. 5B. (C) Uncropped blot related to Fig. 6A. (D) Uncropped blots related to related to Fig. 6B. Inset demonstrates the presence of r16F in IP experiment shown in Fig. 6B. It shows re-probing of IP blot with anti-hTMEM16F antibody (@h16F diluted at 1:1,000) after stripping.
